# miR-7-5p Affects Brain Edema After Intracerebral Hemorrhage and Its Possible Mechanism

**DOI:** 10.3389/fcell.2020.598020

**Published:** 2020-12-16

**Authors:** Xiqian Chen, Shuwen Deng, Qiang Lei, Qiang He, Yijun Ren, Yiliu Zhang, Jingjing Nie, Wei Lu

**Affiliations:** Department of Neurology, The Second Xiangya Hospital, Central South University, Changsha, China

**Keywords:** AQP4, brain edema, intracerebral hemorrhage, PI3K, Akt, MiR-7-5p

## Abstract

**Objective:** To explore the relationship between miR-7-5p and brain edema after intracerebral hemorrhage and the role of butylphthalide (NBP) in brain edema after intracerebral hemorrhage.

**Method:** Routine blood testing, C-reactive protein results, and computed tomography data were collected 1, 7, and 14 days after intracerebral hemorrhage in six patients. Levels of MMP-9, ZO-1, occludin, IL-6, TNF-α, and miR-7-5p were detected in each patient's serum. Sixty male Sprague–Dawley rats were randomly divided into sham operation, intracerebral hemorrhage, and NBP treatment groups. Dry–wet weight was used to assess brain edema, and Evans blue staining was used to assess the permeability of the blood–brain barrier. Expression levels of IL-6, TNF-α, ZO-1 and occludin, PI3K, AKT, p-AKT, AQP4, and miR-7-5p were analyzed in the rat brains.

**Result:** The blood neutrophil–lymphocyte ratio (NLR) on day 1 was associated with the area of brain edema on day 7. The expression of miR-7-5p decreased after intracerebral hemorrhage, and as a result, the inhibition of the PI3K/AKT pathway was weakened. The decreased inhibition of the PI3K/AKT pathway resulted in an increase in AQP4 expression, which further aggravated brain edema. NBP can upregulate the expression of miR-7-5p, affecting these pathways to reduce brain edema.

**Conclusion:** After intracerebral hemorrhage, miR-7-5p expression in brain tissue is reduced, which may increase the expression of AQP4 by activating the PI3K/AKT pathway. NBP can inhibit this process and reduce brain edema.

## Introduction

Intracerebral hemorrhage (ICH) is an important cause of disability and death in the elderly. The formation of brain edema plays an important role in the neurological deficit caused by ICH. The pathophysiological mechanisms of brain edema after ICH are not fully understood, and the factors involved are complex and diverse. Preventing brain edema after ICH is of great significance to the treatment of ICH.

Aquaporins (AQPs) are a family of proteins containing more than a dozen subtypes, and their main role is to promote the transport of water in tissues and cells (Hatakeyama et al., 2001). In the brain, AQP4 is the most abundant AQP (Zhang C. et al., 2019). A large number of studies have shown that elevated AQP4 expression is the main cause of brain edema caused by brain injury (Lo Pizzo et al., 2013; Khalaf et al., 2018; Glober et al., 2019; Zhang Y. et al., 2019; Clement et al., 2020). However, there have been several studies that found that AQP4 expression may be regulated by the PI3K/AKT signaling pathway (Kapoor et al., 2013; Chu et al., 2017; Zhao et al., 2017; Song et al., 2018). The PI3K/AKT signaling pathway is involved in the regulation of various cell functions, such as cell proliferation, differentiation, apoptosis, and glucose transport (Guo et al., 2019). There are various molecules that affect the activity of the PI3K/AKT pathway. Recently, a study has found that a microRNA, miR-7-5p, can affect the activity of the PI3K/AKT pathway (Shukla et al., 2018).

MicroRNAs (miRNAs) are a class of endogenous non-coding RNAs found in eukaryotes with regulatory functions (Ahadi, 2020). They guide the silencing complex to degrade the target mRNA or suppress the translation of the target mRNA (Ahadi, 2020). In previous studies, miR-7-5p has shown a tumor-suppressing effect and is downregulated in many cancers, including melanoma and glioma. miR-7-5p can reduce tumor cell proliferation, growth, migration, and invasion (Giles et al., 2013; Liu et al., 2015; Cheng et al., 2017; Shukla et al., 2018; Hu et al., 2019). In the study of pulmonary edema, it is speculated that miR-7-5p can affect the activity of the mTORC2/SGK-1 pathway to regulate the expression of sodium channels, which affects the formation of pulmonary edema (Qin et al., 2016). mTORC2 belongs to the PI3K-related protein kinase family, participating in regulating AKT signaling pathways (Xu et al., 2020). In their study, Qin et al. suggest that miR-7-5p is related to pulmonary edema (Qin et al., 2016); therefore, we considered whether miR-7-5p can affect brain edema. Previous studies found that miR-7-5p affected PI3K/AKT pathway activity, and the PI3K/AKT pathway regulates AQP4 expression. However, no study has explored the relationship between miR-7-5p and AQP4. Therefore, we decided to explore this relationship to determine if miR-7-5p can affect AQP4 expression and the development of brain edema after ICH.

Butylphthalide (NBP) has a series of pharmacological mechanisms such as rebuilding microcirculation, protecting mitochondria, inhibiting inflammatory responses, and inhibiting neuronal apoptosis (Chong and Feng, 1997; Liu et al., 2007; Zhao et al., 2016; Chen X. Q. et al., 2019; Chen Y. et al., 2019). Currently, it is mainly used for the treatment of ischemic stroke, but several studies have shown that NBP can reduce brain edema after brain injury (Chong and Feng, 1999; Bi et al., 2016; Feng et al., 2018; Chen X. Q. et al., 2019). It has been found that NBP can also activate the PI3K/AKT signaling pathway (Chen et al., 2018; Wang et al., 2018). Interestingly, previous animal studies by our research group found that NBP can reduce brain edema after ICH, but the specific mechanism was not clear. Therefore, in this study, we aimed to further explore the specific mechanism of NBP in reducing brain edema after ICH. In view of the close relationship between AQP4 and brain edema found in previous experiments, we focused our research on whether the mechanism of NBP in reducing brain edema after ICH involves AQP4 expression and miR-7-5p expression. Therefore, we chose NBP as an intervention agent to provide a new therapeutic entry point for clinical treatment of brain edema after ICH.

## Materials and Methods

### Ethics Statement

The study was approved by local ethics committees. All study participants or their legally authorized representatives signed written informed consent.

### Patients

#### Collection of Human Serum Samples and Clinical Case Data

Blood samples were collected from patients with ICH from 2019 to 2020 at the second Xiangya Hospital. The criteria for inclusion of patients with ICH were as follows: (1) 18–80 years of age; (2) meeting the diagnostic criteria for ICH; (3) enrolled patients had spontaneous ICH; (4) hematoma volume <30 ml; (5) there were no other factors that could cause brain edema; and (6) treatments were the same between patients. The exclusion criteria were as follows: (1) rebleeding after admission; (2) hematoma spread into the ventricle; (3) history of stroke such as cerebral hemorrhage, cerebral infarction, or mRS score 0 before onset; (4) tumors in other systems or autoimmune diseases; (5) patients with systemic or local infection; and (6) poor blood glucose control during hospitalization. A total of 10 patients were enrolled at the beginning of the study, four of whom dropped out due to early discharge. Clinical records collection, auxiliary routine blood examination, and C-reactive protein (CRP) analysis (from 6 ml of blood) were performed on days 1, 7, and 14 after onset of ICH.

#### Patient's Neurological Deficit Score

The National Institutes of Health Stroke Scale (NIHSS) was used to evaluate neurological deficits on days 1, 7, and 14. Higher scores indicate more severe neurological deficits, and lower scores indicate less neurological impairment.

#### ICH Volume and Area of the Edema Band Measured on Computed Tomography

Head computed tomography (CT) examinations were performed on days 1, 7, and 14 after onset,

$$\begin{array}{l} \text{ICH volume}\left(\text{mL}\right)=\text{longest axis }\left(\text{cm}\right)\text{of the largest area of the lesion}\\ \;\times \text{short axis of the largest area of the lesion }\left(\text{cm}\right)\\ \;\times \text{layer spacing }(\text{cm})\;\times 0.5 \end{array}$$

$$\begin{array}{l} \text{The area of the edema band around the hematoma at the largest level}\\ \;(\text{cm}2)=\text{length of the outer diameter of the edema band}\\ \;\times \text{width}-\text{length of the inner diameter of the edema band}\\ \;\times \text{width}. \end{array}$$

#### Enzyme-Linked Immunosorbent Assay

Serum levels of MMP-9, ZO-1, occludin, TNF-α, and IL-6 were detected using commercially available MMP-9 (Proteintech, Rosemont, IL; cat. no. KE00164, lot 40000599), ZO-1 (Wuhan Huamei Biotech, Wuhan, China; cat. no. CSB-EL016263HU, lot C10035624), occludin (Wuhan Huamei Biotech, Wuhan, China; cat. no. CSB-E13916h, lot O20316324), TNF-α (Proteintech; cat. no. KE00068, lot 40000504), and IL-6 (Proteintech; cat. no. KE00139, lot 40000524) enzyme-linked immunosorbent assay (ELISA) kit according to manufacturers' instructions.

#### qRT-PCR

Serum levels of miR-7-5p were detected by quantitative real-time polymerase chain reaction (qRT-PCR) on a PikoReal 96 Real-Time PCR system (Thermo Fisher Scientific, Waltham, MA). The reaction conditions were pre-denaturation at 95°C for 10 min, cycling for 40 times at 95°C for 15 s and 60°C for 30 s, and final extension at 72°C for 5 min. The annealing temperature was 58.5°C. Target gene sequences were searched on the National Center of Biotechnology Information database, and primers were designed using Primer5 software and synthesized by Sangon Biotech (Shanghai, China). U6 was used as an internal control for miR-7-5p expression. The primers were used as follows: U6 (Forward: 5′-CTCGCTTCGGCAGCACA-3′; Reverse: 5′-AACGCTTCACGAATTTGCGT-3′), miR-7-5p (Forward: 5′-CGCAGTGGAAGACTAGTGA-3′; Reverse: 5′-CAGGTCCAGTTTTTTTTTTTTTTTAAC-3′). qRT-PCR analysis was performed using the delta-delta Ct method. Samples from the day 7 group were used as control samples.

### Animals

#### Animal Grouping

We purchased 75 healthy male Sprague–Dawley (SD) rats (Hunan SJA Laboratory Animal Company, Changsha, China), aged 8 weeks, weighing between 250 and 300 g, and kept them in standard animal room polypropylene cages. Fifteen rats died due to model failure or other reasons during the experiment, and 60 rats were successfully modeled and survived to the end of the observation. Experiment grouping includes a sham operation group; 1-, 3-, and 7-day ICH group; and an NBP intervention group. Each group had 12 rats. The rats in the NBP group were given intraperitoneal injection of 2 mg/kg NBP (CSPC NBP Pharmaceutical Company, Shijiazhuang, China) for 3 days. The sham operation group and the ICH group were given intraperitoneal injection of 1 ml/kg of 0.9% NaCl every day until the day before sacrifice.

#### ICH Model

Anesthetized rats were fixed in an animal's skull stereotactic instrument and the scalp was cut. Using the anterior fontanel as the origin, the injection point was 3 mm to the right, 1 mm to the front, and 5 mm deep (to the caudate nucleus). The skull was perforated with a bone drill, the collagenase was slowly injected, and the needle was left for 10 min after insertion, before it was slowly withdrawn. Bone wax was used to close the bone holes, and the skin was sutured.

#### Evaluation of Neurological Scores

Neurological Severity Scores were used to evaluate neurological deficits on days 1, 3, and 7. Rats with a neurological defect score of 1–3 were included in the study.

#### Measurement of Brain Edema

Brains were harvested directly after decapitation. The olfactory bulb and lower brain stem were removed, the left and right cerebral cortex were separated, and the basal nucleus and left and right cerebellum was separated. The right side of the cerebral cortex, the basal nucleus, and the left and right cerebellum were weighed immediately, and placed in a 60°C electric oven (Beijing Yongguangming Medical Instrument Company, Beijing, China) to bake to constant weight. The dried brains were then quickly weighed. Brain water content was calculated as follows: (wet weight–dry weight)/wet weight.

#### Evans Blue Staining

Evans blue solution (1%, Sigma-Aldrich, St. Louis, MO; cat. no. E2129, lot MKCB2532V) was injected into the tail vein 3 h before rats were sacrificed. The injection volume was 1 ml per 100 g. Rats were anesthetized before harvesting brain tissue. Rats were perfused via the heart with 0.9% physiological saline, and harvested brain tissue was fixed with 4% paraformaldehyde. After the whole brain was fixed, the whole brain (except the cerebellum) was cut into about 2-mm slices. For quantitative measurement, brain hemispheres were homogenized in 3 ml of N,N-dimethylformamide (Sigma-Aldrich) and centrifuged. The supernatant was analyzed by spectrophotometry at 620 nm.

#### Immunohistochemistry

Immunohistochemistry was used to detect AQP4 in rat brain tissue. The specimens were embedded in paraffin and sectioned. Slices were immersed in 0.01 M citrate buffer (Wellbio, pH 6.0), heating to boiling in a microwave oven (Midea Foshan, China; cat. no. MM721AAU-PW), and cooling to room temperature. After cooling, slices were washed with 0.01 M PBS (Wellbio, pH 7.2–7.6), incubated in 3% H_2_O_2_ at room temperature for 16 min to inactivate endogenous enzymes, and then rinsed with PBS. AQP4 antibody (Proteintech; cat. no. 16473-1-AP, anti-rabbit, dilution 1:200) was added to slices and incubated at 4°C overnight. After washing with PBS, 50–100 μl of secondary antibody [Proteintech; cat. no. SA00001-2, horseradish peroxidase (HRP)-conjugated goat anti-rabbit IgG, dilution 1:6,000] was added and incubated at 37°C for 30 min. Slices were then again washed in PBS and stained with DAB. Positive staining of cells was seen as yellow or brownish yellow (dark to brown). An ordinary computer was used to acquire images using ImageJ (National Institutes of Health, Bethesda, MD).

#### Western Blot

Western blotting was used to detect PI3K, AKT, p-AKT, AQP4, ZO-1, occludin, IL-6, and TNF-α in rat brain tissue. Brain tissue proteins were extracted and transferred to a polyvinylidene fluoride filter membrane by electrophoresis. The membrane was then immersed in isotonic buffered saline solution containing 5% skimmed milk powder and blocked at room temperature. The membrane was then incubated in diluted primary antibodies [PI3K (Proteintech; cat. no. 20584-1-AP, anti-rabbit, dilution 1:500), AKT (Proteintech cat. no. 10176-2-AP, anti-rabbit, dilution 1:1,000), p-AKT (Abcam, Cambridge, UK; cat. no. ab192623, anti-rabbit, dilution 1:1,000), AQP4 (Proteintech; cat. no.16473-1-AP, anti-rabbit, dilution 1:1,000), ZO-1 (Proteintech; cat. no. 21773-1-AP, anti-rabbit, dilution 1:4,000), occludin (Proteintech; cat. no. 13409-1-AP, anti-rabbit, dilution 1:2,000), IL-6 (Proteintech; cat. no. 21865-1-AP, anti-rabbit, dilution 1:1,000), and TNF-α (Proteintech; cat. no. 17590-1-AP, anti-rabbit, dilution 1:500)] at 4°C overnight. After washing with isotonic buffered saline solution, membranes were incubated with a secondary antibody (Proteintech; cat. no. SA00001-1, HRP-conjugated goat anti-mouse IgG, dilution 1:5,000; Proteintech; cat. no. SA00001-2, HRP-conjugated goat anti-rabbit IgG, dilution 1:6,000) for 1 h at room temperature and then washed with isotonic buffered saline solution for color development. The film was developed in an X-ray film cassette. β-actin (Proteintech; cat. no. 66009-1-Ig, anti-mouse, dilution 1:5,000) was used as an internal reference.

#### qRT-PCR

miR-7-5p in the rat brain tissue was detected by qRT-PCR (PikoReal 96, Thermo Fisher Scientific). Trizol (Thermo Fisher Scientific, cat. no. 15596026) was used to extract total RNA from brain tissue. The reaction conditions were pre-denaturation at 95°C for 10 min, cycling 40 times at 95°C for 15 s and 60°C for 30 s, and final extension at 72°C for 5 min. The annealing temperature was 58.5°C. Target gene sequences were searched on the National Center of Biotechnology Information database, and the primers were designed by Primer5 software and synthesized by Sangon Biotech. 5S was used as an internal control for miR-7-5p expression. The primers were used as follows: 5S (Forward: 5′-GCCTACAGCCATACCACCCGGAA-3′; Reverse: 5′-CCTACAGCACCCGGTATCCCA-3′), miR-7-5p (Forward: 5′-ACACTCCAGCTGGGTGGAAGACTAGTGATTTT-3′; Reverse: 5′-CTCAACTGGTGTCGTGGAGTCGGCAATTCAGTTGAGACAACAAA-3′). qRT-PCR analysis was performed using the delta-delta Ct method. The sham operation group was used as control samples.

### Statistical Analysis

SPSS 21.0 (IBM, Armonk, NY) was used for statistical analysis. The data were expressed as mean ± standard deviation. The multi-sample mean was analyzed by variance. Pairwise comparison between groups was performed by the least significant difference *t*-test. Non-normally distributed data were analyzed using the Mann–Whitney *U* test. Correlations were analyzed by linear correlation analysis. *P*-values < 0.05 were considered statistically significant. Bar graphs were generated with GraphPad Prism (version 5.01; GraphPad Software, San Diego, CA).

## Results

### Patients

#### Analysis of Clinical Data of Patients

The patient group consisted of two women and four men with an average age of 62.7 years (range: 58–87 years). The NIHSS score was significantly higher (*P* < 0.05) and neurological deficits were worse on day 7 after ICH compared to day 1. Similarly, the NIHSS score on day 14 after ICH was lower than that on days 7 (*P* < 0.05) and 1 (*P* < 0.05). In addition, the blood neutrophil–lymphocyte ratio (NLR) on day 1 was associated with the area of brain edema on day 7 after ICH (*r* = 0.879, *P* < 0.05) (Supplementary Figure 1). The NLR on day 7 after ICH was lower than that on day 1, but the difference was not significant (*P* = 0.074). The NLR on day 14 after ICH did not differ significantly from that on day 7 (*P* = 0.13), but it was significantly lower than that on day 1 (*P* < 0.05) (Supplementary Figure 2A). The CRP level on day 7 after ICH was not significantly different than that on day 1 (*P* = 0.062), but the CRP level on day 14 after the ICH was significantly lower than that on day 1 or 7 (*P* < 0.05) (Supplementary Figure 2B). The volume of hematoma as shown by head CT on day 7 was less than that on day 1 (*P* < 0.05). Similarly, the area of edema around the hematoma on day 7 was larger than that on day 1 (*P* < 0.05). The volume of hematoma on day 14 was lower than that on day 7 (*P* < 0.05). The area of edema around the hematoma on day 14 was lower than that on day 7 (*P* < 0.05). The volume of hematoma on day 14 was significantly lower than that on day 1 (*P* < 0.05). The area of edema around hematoma on day 14 of ICH was lower than that on day 1, but the difference was not statistically significant (Table 1).

**Table 1:** NIHSS score, hematoma volume, brain edema, NLR, and CRP on days 1, 7, and 14 after intracerebral hemorrhage of patients.

**Group**	**ICH 1d**	**ICH 7d**	**ICH 14d**
NHISS	8.50 ± 2.07	10.00 ± 2.53^*^	7.17 ± 1.47^*^^#^
Hematoma volume (ml)	14.66 ± 3.04	11.08 ± 2.97^*^	7.52 ± 2.85^*^^#^
Edema area (cm^2^)	2.88 ± 0.80	6.63 ± 1.47^*^	2.53 ± 0.29^*^
NLR	4.24 ± 2.25	2.22 ± 0.28	1.94 ± 0.16^#^
CRP (mg/L)	9.77 ± 4.77	7.03 ± 2.00	3.95 ± 1.34^*^^#^

* P < 0.05, for ICH 7d group vs. ICH 1d group or ICH 14d group vs. ICH 7d group;

# *P < 0.05, for ICH 14d group vs. ICH 1d group*.

*ICH, intracerebral hemorrhage; NIHSS, National Institutes of Health Stroke Scale; NLR, neutrophil–lymphocyte ratio; CRP, C-reactive protein*.

#### Dynamic Changes in MMP-9, ZO-1, Occludin, IL-6, and TNF-α Levels After ICH

Serum MMP-9 levels on day 7 after ICH did not differ significantly from those on day 1, but the levels on day 14 were significantly lower than those on day 1 or 7 (*P* < 0.05) (Supplementary Figure 3A). Serum levels of tight junction proteins ZO-1 and occludin were significantly higher on day 7 than on day 1 (*P* < 0.05), and their levels on day 14 remained significantly higher than those on day 1 (*P* < 0.05) (Supplementary Figure 3B). The levels of ZO-1 and occludin on day 14 were not significantly different than those on day 7 (Supplementary Figure 3B). The serum IL-6 level was significantly lower on day 7 than on day 1 (*P* < 0.05), and it remained significantly lower on day 14 than on day 1 (*P* < 0.05); but there was no significant difference in levels between days 7 and 14 (Supplementary Figure 3C). The level of TNF-α was higher on day 7 than on day 1, but this was not statistically significant. The level of TNF-α on day 14 was not significantly different from days 1 or 7 (Supplementary Figure 3C).

#### Serum Mir-7-5P Levels After ICH

**Figure 1:**
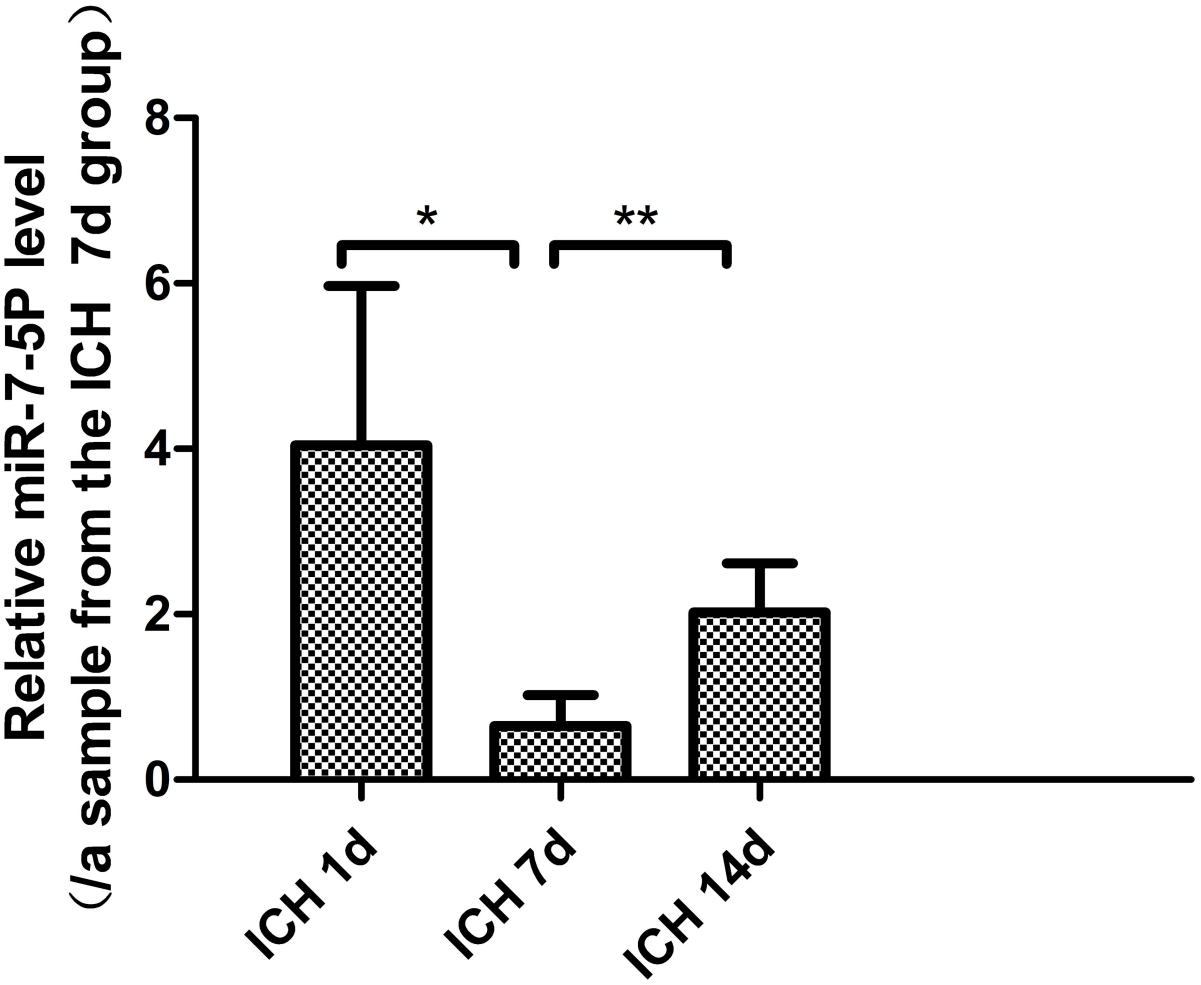
miR-7-5p expression in patients with intracerebral hemorrhage on day 7 was significantly lower than that on day 1, and partially recovered on day 14. *P < 0.05, **P < 0.01.

The level of miR-7-5p on day 7 after ICH was significantly lower than that on day 1 (*P* < 0.05), and it partially recovered on day 14. The miR-7-5p level on day 14 was significantly higher than that on day 7 (*P* < 0.05), but it was not significantly different from day 1 (Figure 1).

### Animals

#### NBP Improved Neurological Deficits in Rats

The neurological deficit score was higher on day 3 after ICH than on day 1 (*P* < 0.05), but the neurological deficit score was not significantly different between days 3 and 7 (*P* > 0.05). The neurological deficit improved after 3 days of NBP intervention (*P* < 0.05) (Table 2).

**Table 2:** Nerve function defect and brain water content in each group of rats.

**Group**	**Sham**	**ICH 1d**	**ICH 3d**	**ICH 7d**	**NBP**
NSS	0	1.00 ± 0.00^*^	1.83 ± 0.75^*^	1.67 ± 0.52^*^	1.17 ± 0.41^#^
Water content (%)	77.37% ± 0.94%	81.51% ± 1.23%^*^	81.65% ± 0.84%^*^	80.09% ± 1.18%^*^	78.77% ± 0.51%^#^

* P < 0.05, for each timepoint group of ICH vs. sham group;

# *P < 0.05, for NBP intervention group vs. ICH 3d group*.

*ICH, intracerebral hemorrhage; NBP, butylphthalide; NSS, Neurological Severity Score*.

#### NBP Reduced Brain Edema

The brain tissue water content in the ICH group increased significantly (*P* < 0.05). Obvious edema was observed on day 1 after ICH, and it remained obvious for 7 days. The brain water content decreased in the NBP group (*P* < 0.05). We speculated that NBP could reduce brain edema after ICH and thus play a protective role against ICH (Table 2).

#### Evans Blue Staining Suggested That NBP Reduced Blood–Brain Barrier Permeability

**Figure 2:**
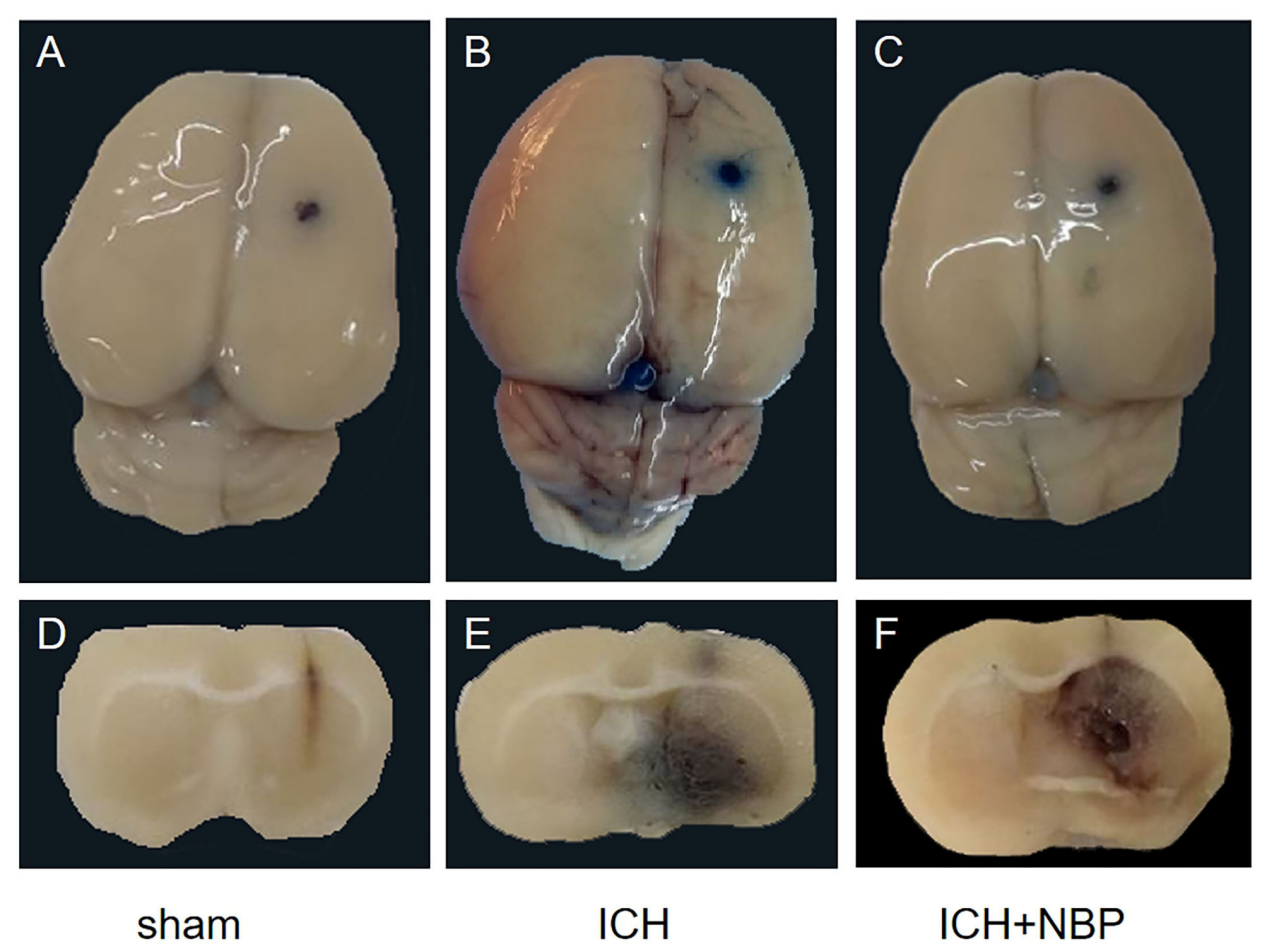
Figures **(A–C)** showed the extravasation of Evans Blue on the surface of the rat brain tissue, and figures **(D–F)** showed the extravasation of Evans Blue around the hematoma in the brain tissue section of the rats. It could be seen that extravasation of Evans blue is reduced after NBP intervention.

**Figure 3:**
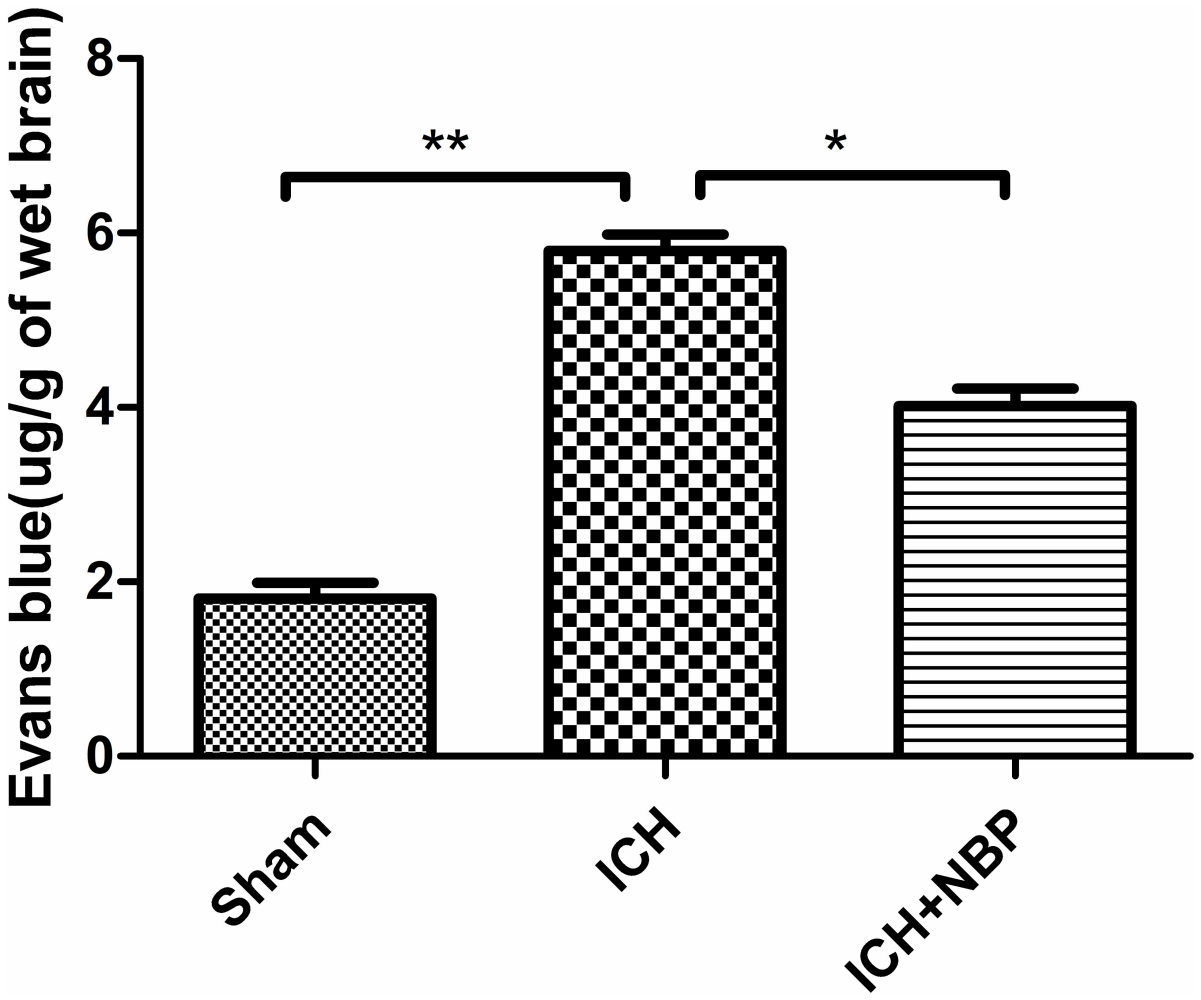
Extravasation of Evans blue reduced after NBP intervention. *P < 0.05, **P < 0.01.

Extravasation of Evans blue around the brain hematoma and the brain surface increased after ICH, and the extravasation of Evans blue decreased after the addition of NBP (Figures 2, 3).

#### AQP4 Expression Was Upregulated in Brain Tissue After ICH and Decreased After Intervention With NBP

**Figure 4:**
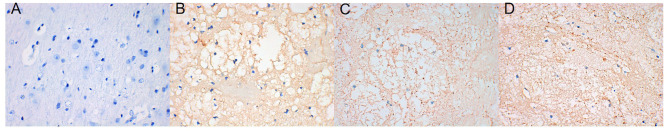
AQP4 increased significantly in brain tissue on days 1, 3, and 7 after intracerebral hemorrhage (the yellow-dyeing part indicated by the arrow was where AQP4 located, and the blue part was the nucleus). **(A)** Sham group. **(B)** ICH 1d group. **(C)** ICH 3d group. **(D)** ICH 7d group.

**Figure 5:**
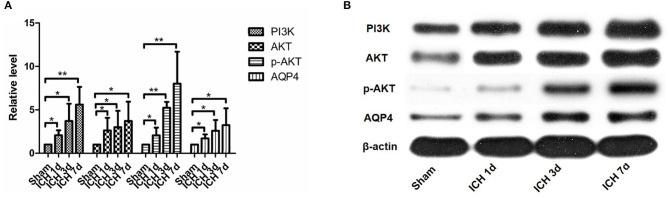
PI3K, AKT, p-AKT, and AQP4 expressions increased after intracerebral hemorrhage. They can be seen to increase significantly within 1 day after intracerebral hemorrhage, and they continued to increase within 7 days after intracerebral hemorrhage **(A,B)**. *P < 0.05, **P < 0.01.

**Figure 6:**
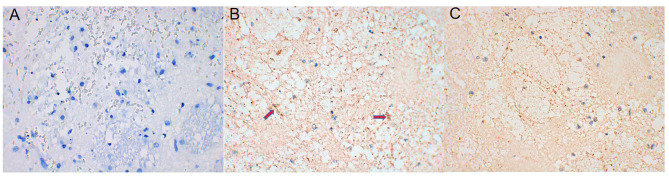
AQP4 expression decreased after NBP intervention (the yellow-dyeing part indicated by the arrow was where AQP4 located, and the blue part was the nucleus). **(A)** Sham group. **(B)** ICH 3d group. **(C)** ICH 3d + NBP group.

**Figure 7:**
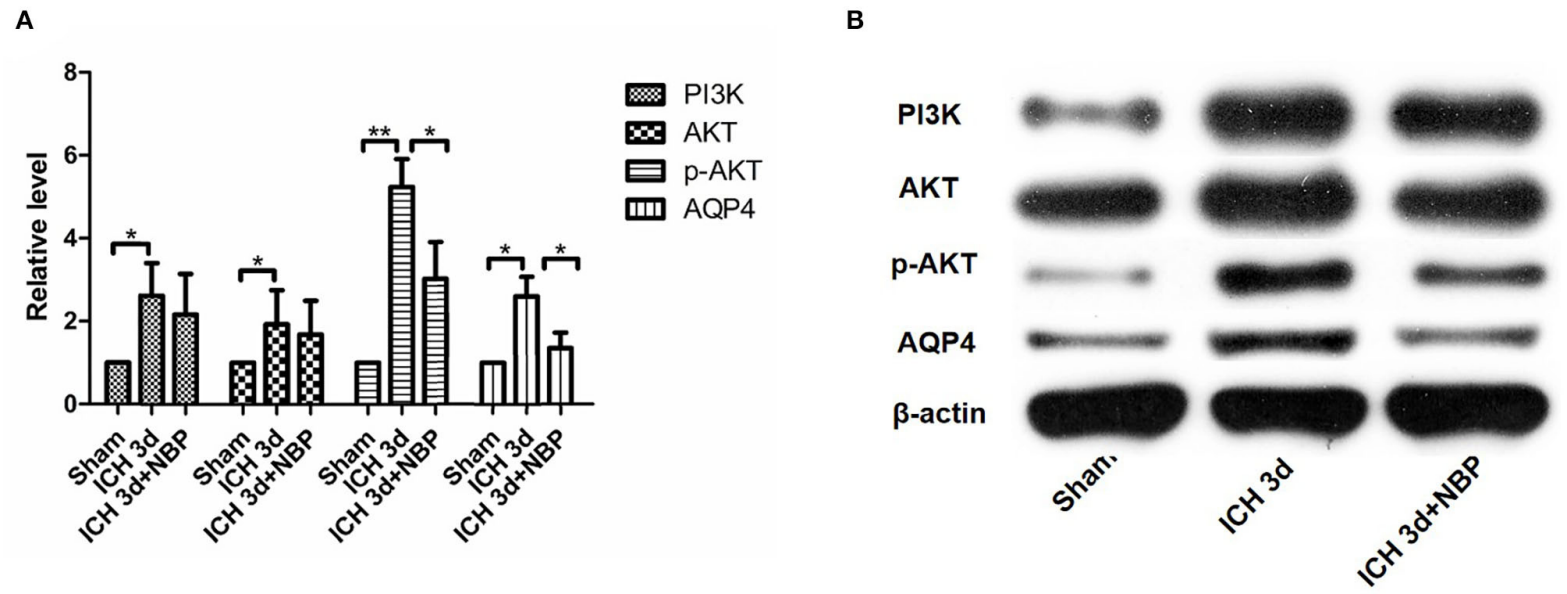
PI3K did not change significantly in the NBP intervention group **(A,B)**. AKT expression did not change significantly after the intervention with NBP **(A,B)**. However, p-AKT expression decreased after the intervention with NBP **(A,B)**. AQP4 expression decreased in the NBP intervention group **(A,B)**. *P < 0.05, **P < 0.01.

AQP4 expression around the hematoma of the brain tissue was higher on days 1, 3, and 7 in the ICH group compared to the sham operation group (*P* < 0.05) (Figures 4, 5). However, AQP4 expression around the hematoma of the rat brain tissue decreased after 3 days of intervention with NBP (*P* < 0.05) (Figures 6, 7).

#### TNF-α and IL-6 Expression Increased and ZO-1 and Occludin Expression Decreased After ICH. NBP Downregulated the Expression of TNF-α and IL-6 and Upregulated the Expression of ZO-1 and Occludin

The expression of TNF-α and IL-6 in brain tissues increased significantly after ICH (*P* < 0.05) (Supplementary Figure 4). They began to increase on day 1 after ICH and reached their peaks on day 3. They remained at a high level on day 7 (Supplementary Figure 4). However, ZO-1 and occludin levels in the ICH group were lower than in the sham operation group (*P* < 0.05) (Supplementary Figure 4). The decreases in ZO-1 and occludin levels were most obvious on day 1, and they remained significantly low on day 3; they gradually recovered afterwards (Supplementary Figure 4). The expression of TNF-α and IL-6 was lower and that of ZO-1 and occludin was higher in the NBP group than in the ICH model group (both *P* < 0.05) (Supplementary Figure 5).

#### PI3K/AKT Pathway Activation After ICH. NBP Inhibits the Activation of the PI3K/AKT Pathway After ICH

PI3K expression in the brain tissue of rats in the ICH group was significantly higher than that in the sham operation group (*P* < 0.05). PI3K levels increased on day 1 after ICH and continued to increase within 7 days after ICH (Figure 5). AKT levels increased significantly after ICH (*P* < 0.05) from days 1 to 7 after ICH (Figure 5). p-AKT levels were also significantly higher in the brain tissue in the ICH group than in the sham operation group, and this trend was consistent with that of AKT (Figure 5). In addition, the levels of p-AKT/AKT in the brain tissue in the ICH group were significantly higher than that in the sham operation group (*P* < 0.05). In the NBP group, neither PI3K nor AKT levels in the brain tissue changed significantly (Figure 7). However, p-AKT levels were lower in the brain tissue of the NBP group than that of the ICH model group (*P* < 0.05) (Figure 7). The p-AKT/AKT ratio in the NBP group was lower than that in the ICH group (*P* < 0.05).

#### Expression of Mir-7-5p Significantly Decreased and NBP Reversed the Downregulation of Mir-7-5p Expression After ICH

**Figure 8:**
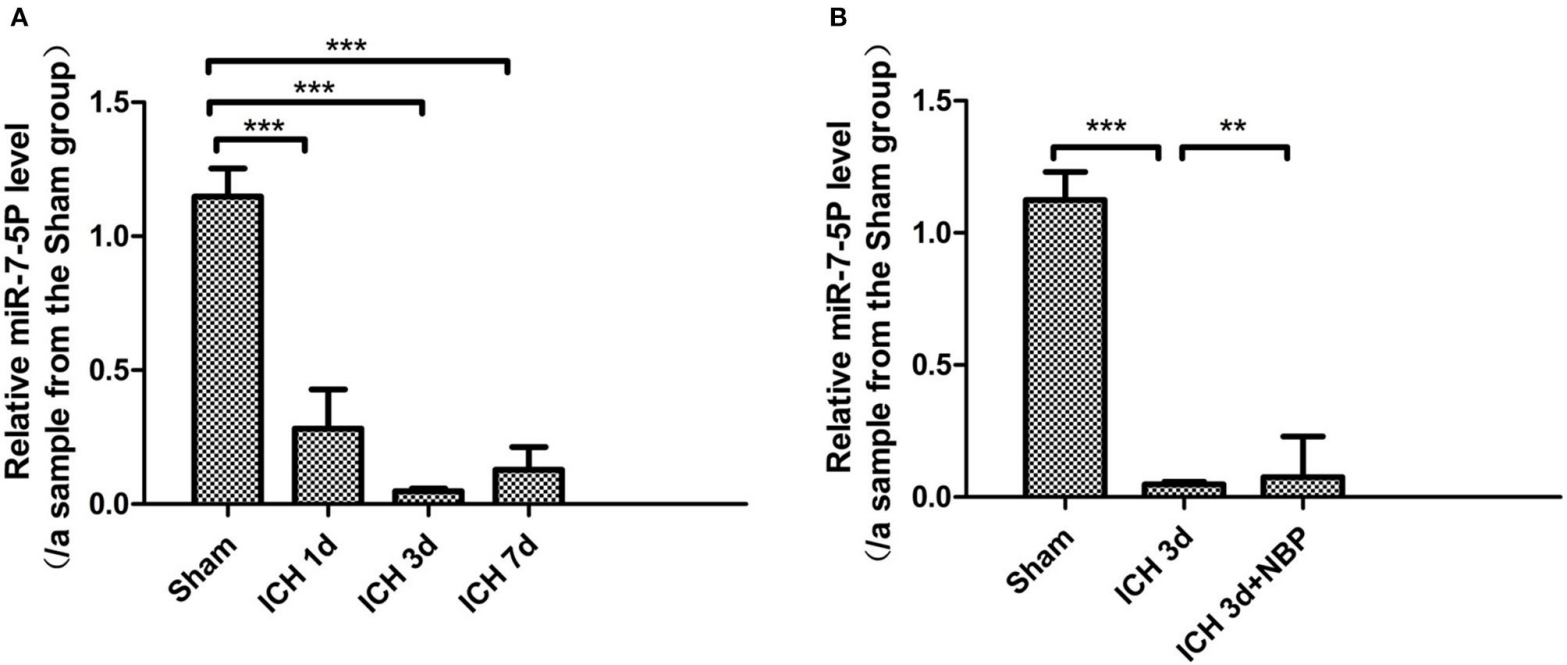
miR-7-5p expression decreased after intracerebral hemorrhage **(A)**, and miR-7-5P expression was the lowest on day 3 after intracerebral hemorrhage **(A)**. miR-7-5p expression increased after the intervention of NBP **(B)**. *P < 0.05, **P < 0.01, ***P < 0.001.

We detected that the expression of miR-7-5p was significantly downregulated in the brain tissue on the 1st, 3rd, and 7th days after ICH (*P* < 0.05), among which the decrease in miR-7-5p was the most obvious on the 3rd day after ICH (Figure 8A); the expression of miR-7-5p was recovered partially after administering NBP intervention (*P* < 0.05) (Figure 8B).

## Discussion

The pathogenesis of brain edema after ICH is not completely clear. The currently known pathogenesis includes blood clot retraction and accumulation of serum proteins around the blood clot, which causes brain edema; hemoglobin and its degradation products and red blood cell components can also cause edema (Wilkinson et al., 2018). The destruction of endothelial cells and the blood–brain barrier (BBB) by various factors, including thrombin, causes vascular edema (Wilkinson et al., 2018). In addition, expression of AQPs, inflammatory cytokines, and other factors play important roles in the development of brain edema after ICH (Wilkinson et al., 2018). At present, an increasing number of studies have begun to explore the role of miRNAs in ICH. miRNAs have also been found to be abnormally expressed in rat brains with ICH, and it has been found to be involved in pathophysiological processes like neuronal apoptosis and brain edema after ICH (Shang et al., 2019). Previous studies have confirmed that miR-7-5p is expressed in normal brain tissue, and some researchers believe that research on miR-7-5p may yield valuable insights into brain diseases (Liu et al., 2014). Current research on miR-7-5p in relation to brain tissue diseases is limited to glioma and ischemic brain injury (Liu et al., 2014; Xu et al., 2019). In ischemic brain injury, miR-7-5p can also inhibit inflammatory responses and oxidative stress to play a protective role in the brain (Xu et al., 2019). These studies have shown that miR-7-5p plays multiple roles in brain diseases. The study by Qin et al. suggests that miR-7-5p affects the formation of pulmonary edema; therefore, we considered whether miR-7-5p could also affect brain edema after ICH (Qin et al., 2016). Previous studies have found that miR-7-5p affects PI3K/AKT pathway activity, and PI3K/AKT pathway regulates AQP4 expression, but there have been no studies exploring the relationship between miR-7-5p and AQP4. This study aimed to explore whether miR-7-5p can affect AQP4 expression after ICH through the PI3K/AKT pathway and to explore whether these processes ultimately affect the development of brain edema after ICH. Furthermore, we observed whether NBP can interfere with this process.

At present, it is known that besides AQP4 expression, factors affecting brain edema after ICH usually include damage to the BBB and inflammatory reactions. MMP-9, ZO-1, and occludin are closely related to the BBB, while TNF-α, IL-6, NLR, and CRP are closely related to inflammation after ICH. As AQP4 cannot be detected in the serum of patients with ICH, we examined MMP-9, ZO-1, occludin, TNF-α, IL-6, NLR, and CRP in the serum of patients with ICH. We wanted to know if miR-7-5p is also associated with MMP-9, ZO-1, occludin, TNF-α, IL-6, NLR, and CRP and if it jointly affects brain edema. Studies have found that NLR may be related to the edema around the hematoma in patients with ICH (Gusdon et al., 2017; Fonseca et al., 2020). The baseline NLR can predict the occurrence and development of brain edema because a high NLR may result in neutrophil-induced neurotoxicity, increased capillary permeability, destruction of the BBB, and cell swelling (Zou et al., 2019). In our study, the NLR on day 1 was related to the area of brain edema day 7 after ICH, which was the most obvious period of brain edema. This finding suggests that precautions are needed to prevent brain edema in patients with a high baseline NLR. NLR had a downward trend within 14 days after ICH, but it was not statistically significant; this may be related to the insufficient number of cases included in this study. The CRP level, an inflammatory index, was decreased on day 14 after ICH, indicating that the body's inflammatory response had been reduced on day 14. Increased levels of IL-6 and TNF-α represent an obvious inflammatory response. Studies on patients with brain injury showed that IL-6 levels were high within a few hours after brain injury and that they gradually decreased after 1–2 days (Woiciechowsky et al., 2002). One study on patients with ICH found that IL-6 and TNF-α levels decreased on day 14 (Jiang et al., 2016). In our study, IL-6 levels decreased on day 7, suggesting that the inflammatory response mediated by IL-6 had gradually reduced on day 7 after ICH. However, the change in TNF-α levels at each timepoint was not obvious, which is inconsistent with previous research; this aspect must be studied further. MMP-9 is an important proteolytic enzyme, and an increase in MMP-9 levels can cause the destruction of the BBB, which aggravates brain edema (Li et al., 2018). Several studies on patients with ICH presented different opinions. One is that MMP-9 starts to increase on day 1 after ICH and reaches a peak on day 3, rising again on day 7 followed by a slow decline for a longer period of time (Alvarez-Sabin et al., 2004). The second opinion is that MMP-9 levels increase significantly 2–6 h after ICH and decrease after 5–10 days (Wu et al., 2010). In our experiment, MMP-9 levels decreased on day 14 after ICH; the BBB damage mediated by MMP-9 significantly decreased on day 14, which is consistent with previously reported findings. ZO-1 and occludin are tight junction proteins that participate in the composition of the BBB. Reduced ZO-1 and occludin levels can cause increased BBB permeability. Recent studies in rodents have generally found that ZO-1 and occludin levels begin to decrease within a few hours after ICH and that the decrease was obvious in ~3 days, followed by a gradual recovery (Keep et al., 2018). In our experiments, the expression levels of ZO-1 and occludin were higher on days 7 and 14 after ICH than on day 1. Based on these findings, we think that the expression of ZO-1 and occludin on day 1 after ICH should also decrease and that the human body starts a series of post-stress repair mechanisms after ICH. Therefore, the rise in ZO-1 and occludin levels on days 7 and 14 indicates that the body has initiated a stress repair mechanism to reduce brain edema. Furthermore, we observed that serum miR-7-5p expression after ICH was the lowest on day 7, which partially recovered on day 14. CT imaging showed that the area of edema was the largest on day 7 and that it decreased on day 14. The trend of change in miR-7-5p levels was opposite to that of brain edema, which suggests that miR-7-5p may be related to the formation of brain edema after ICH. There was no clear relationship between the change in the trend of miR-7-5p levels in serum and changes in MMP-9, ZO-1, occludin, IL-6, TNF-α, NLR, and CRP levels. We obtained preliminary conclusions from serum indicators of clinical patients that miR-7-5p may affect brain edema, but it does not affect MMP-9, ZO-1, occludin, IL-6, TNF-α, NLR, and CRP expression to affect the formation of brain edema. Therefore, the question arises whether there is a relationship between miR-7-5p and AQP4. To resolve this, we constructed a rat model of ICH to observe the expression of miR-7-5p, PI3K/AKT, AQP4 and the above indicators in rat brains.

In our experiments on rats, the most important finding was that miR-7-5p was significantly downregulated in brain tissue after ICH. Second, our study showed that AKT expression in the brain tissues had increased after ICH, as did that of p-AKT and p-AKT/AKT. This indicates the activation of the PI3K/AKT pathway. Therefore, the relationship between the decline in miR-7-5p expression and the activation of the PI3K/AKT pathway after ICH remains to be explored. Several previous studies have shown that miR-7-5p can inhibit the PI3K/AKT pathway in gliomas, colorectal cancer, and cardiomyocyte autophagy diseases (Kefas et al., 2008; Liu et al., 2014, 2018; Shukla et al., 2018; Cao et al., 2019). First, many previous studies on gliomas found that the decline in miR-7-5p expression in gliomas causes the activation of the PI3K/AKT pathway, resulting in increased tumor cell proliferation (Kefas et al., 2008; Liu et al., 2014; Shukla et al., 2018; Xiao, 2019; Yin et al., 2019). Colorectal cancer-related studies revealed that miR-7-5p can inhibit the activity of the PI3K/AKT pathway and inhibit cell proliferation and invasion (Liu et al., 2018). Moreover, miR-7-5p has been shown to inhibit AKT pathway activation in cardiomyocyte autophagy induced by uric acid and sphingomyelin (Cao et al., 2019). These studies suggest that miR-7-5p can inhibit PI3K/AKT activation. Therefore, we believe that the reduction of miR-7-5P expression in the brain tissue after ICH causes the activation of the PI3K/AKT pathway. In our study, we also found that AQP4 expression increased after ICH. AQP4 plays an important role in the development of brain edema. AQP4 knockout can effectively reduce brain edema (Song et al., 2018; Guan et al., 2020). The reduction of AQP4 expression by a hydrogen-rich solution was found to be related to the inhibition of the PI3K/AKT pathway in a rat model of myocardial injury (Song et al., 2018). Chu et al. found that inhibiting the PI3K/AKT pathway can downregulate AQP4 expression in cerebral ischemic models and eventually reduce edema (Chu et al., 2017). In addition, in another study related to the gastrointestinal tract, astragalus and its components regulate the levels of cytokines and gastrointestinal hormones, which trigger a series of pathways and molecular activations, including that of the PI3K-AKT signaling pathway. These eventually activate effector molecules such as AQP4 (Zhao et al., 2017). A study on brain edema secondary to brain trauma found that the phosphorylation of AKT can affect the phosphorylation of Foxo3a and thus affect the expression of AQP4 (Kapoor et al., 2013). Another study on ischemic cerebral perfusion injury in rats demonstrated that PI3K/AKT can regulate AQP4 expression (Chien et al., 2015). In line with reports suggesting that the PI3K/AKT pathway can positively regulate AQP4 expression in myocardial ischemia, brain trauma, and cerebral ischemic diseases (Kapoor et al., 2013; Chien et al., 2015; Chu et al., 2017; Zhao et al., 2017; Song et al., 2018), our study found that both PI3K, AKT, and AQP4 levels increased, indicating that PI3K/AKT activation can also positively regulate the expression of AQP4 after ICH, which affects brain edema. Because miR-7-5p can inhibit the activity of the PI3K/AKT pathway, it can inhibit AQP4 expression by inhibiting the PI3K/AKT pathway and, consequently, brain edema. Therefore, we came to the first conclusion that the expression of miR-7-5p decreased after ICH; as a result, the inhibition of the PI3K/AKT pathway was weakened, and the activation of the PI3K/AKT pathway resulted in an increase in AQP4 expression and AQP4-aggravated brain edema.

The general effect of NBP on vasodilation is limited to microvessels. In the pathogenesis of ICH, vascular rupture usually occurs in large blood vessels; therefore, NBP does not increase the volume of ICH. In addition to the beneficial effects as mentioned before, it has also been found to protect the BBB and reduce brain edema in diseases such as ischemic stroke and concussion injury (Bi et al., 2016; Feng et al., 2018). Based on the mechanism of action of NBP and the pathogenesis of ICH, we think that NBP may be not only safe but also beneficial in the treatment of ICH. Our research group found that NBP can reduce brain edema after ICH, but the specific mechanism was not clear (Qiu et al., 2019). From data analysis of clinical patients, we found a negative correlation between the expression of miR-7-5p and brain edema after ICH. In the brain tissue of rats with ICH, we further found that miR-7-5p affected AQP4 expression and thus affected brain edema. Therefore, we wanted to observe whether the mechanism of NBP in reducing brain edema after ICH involves affecting the expression of miR-7-5p. Therefore, we decided to use NBP as an intervention in animal models. First, our experiments revealed that the neurological deficit scores of rats and the water content of the brain were lower after intervention in the NBP group than in the ICH group. Reduced extravasation of the Evans blue stain was observed, which suggested that NBP can improve the symptoms of nerve defects after ICH by improving the integrity of the BBB and reducing brain edema. NBP has been found in previous studies to inhibit the increase in IL-6 and TNF-α levels and to reduce inflammatory damage (Liao et al., 2018; Yang et al., 2018). NBP was also found to upregulate the expression of ZO-1 and occludin and reduce the BBB permeability (Bi et al., 2016; Ye et al., 2019). In our study, the expression of TNF-α and IL-6 increased significantly after ICH, and the expression of TNF-α and IL-6 also decreased significantly after intervention with NBP. NBP could also inhibit the expression of these inflammatory factors in ICH to protect brain tissue. Second, ZO-1 and occludin decreased after ICH, and the expression of ZO-1 and occludin also increased after the addition of NBP, which suggests that NBP could protect the BBB after ICH. This process can also alleviate the occurrence and development of brain edema. Most importantly, in the NBP group, AQP4 expression in the brain tissue increased after 3 days of intervention, with a simultaneous decrease in the expression of p-AKT and p-AKT/AKT; this indicated a decrease in the activity of the PI3K/AKT pathway. An increase in miR-7-5p expression in the brain tissue was also observed. This suggests that NBP can inhibit the decrease in miR-7-5p levels, thereby increasing miR-7-5p expression after ICH. The increased miR-7-5p expression can inhibit the PI3K/AKT pathway, which causes a decrease in AQP4 levels. The miR-7-5p/PI3K/AKT/AQP4 pathway may be an important mechanism by which NBP reduces brain edema after ICH. Furthermore, the results relating to brain water content, Evans blue staining, and neurological deficit scores in the NBP intervention group also showed that NBP was effective. Therefore, NBP can reduce brain edema and improve prognosis in various ways. Because of ethical issues, NBP has not been clinically used for the treatment of ICH; therefore, NBP has not yet been used for the treatment of patients with ICH.

## Conclusion

miR-7-5p expression decreased in brain tissue after ICH, and miR-7-5p expression was inversely proportional to the degree of brain edema. miR-7-5p may increase AQP4 expression by activating the PI3K/AKT pathway, thereby causing brain edema. NBP can reduce brain edema after ICH by increasing miR-7-5p expression.

## Data Availability Statement

The raw data supporting the conclusions of this article will be made available by the authors, without undue reservation.

## Ethics Statement

The studies involving human participants were reviewed and approved by Ethics Committee of the Second Xiangya Hospital of Central South University. The patients/participants provided their written informed consent to participate in this study. The animal study was reviewed and approved by Ethics Committee of the Second Xiangya Hospital of Central South University.

## Author Contributions

XC wrote the first draft of the report. WL provided statistical and analysis support. QL, SD, QH, YR, YZ, and JN reviewed the study data, reviewed and edited the report, and approved the final version. All authors contributed to the article and approved the submitted version.

## Conflict of Interest

The authors declare that the research was conducted in the absence of any commercial or financial relationships that could be construed as a potential conflict of interest.

## Abbreviations

BBB: blood–brain barrier
CRP: C-reactive protein
CT: computed tomography
ELISA: enzyme-linked immunosorbent assay
ICH: intracerebral hemorrhage
NBP: butylphthalide
NIHSS: National Institutes of Health Stroke Scale
NLR: neutrophil–lymphocyte ratio
miRNAs: microRNAs
qRT-PCR: quantitative real-time polymerase chain reaction
SD: Sprague–Dawley.
