# Supplementary material for: miR-7-5p Affects Brain Edema After Intracerebral Hemorrhage and Its Possible Mechanism
Source: Front Cell Dev Biol. 2020 Dec 16;8:598020. doi: 10.3389/fcell.2020.598020 (PMC7772315; doi:10.3389/fcell.2020.598020)
Supplement: Supplementary file 1 [file Data_Sheet_1.DOCX]

Supplementary Material

## Supplementary Figures
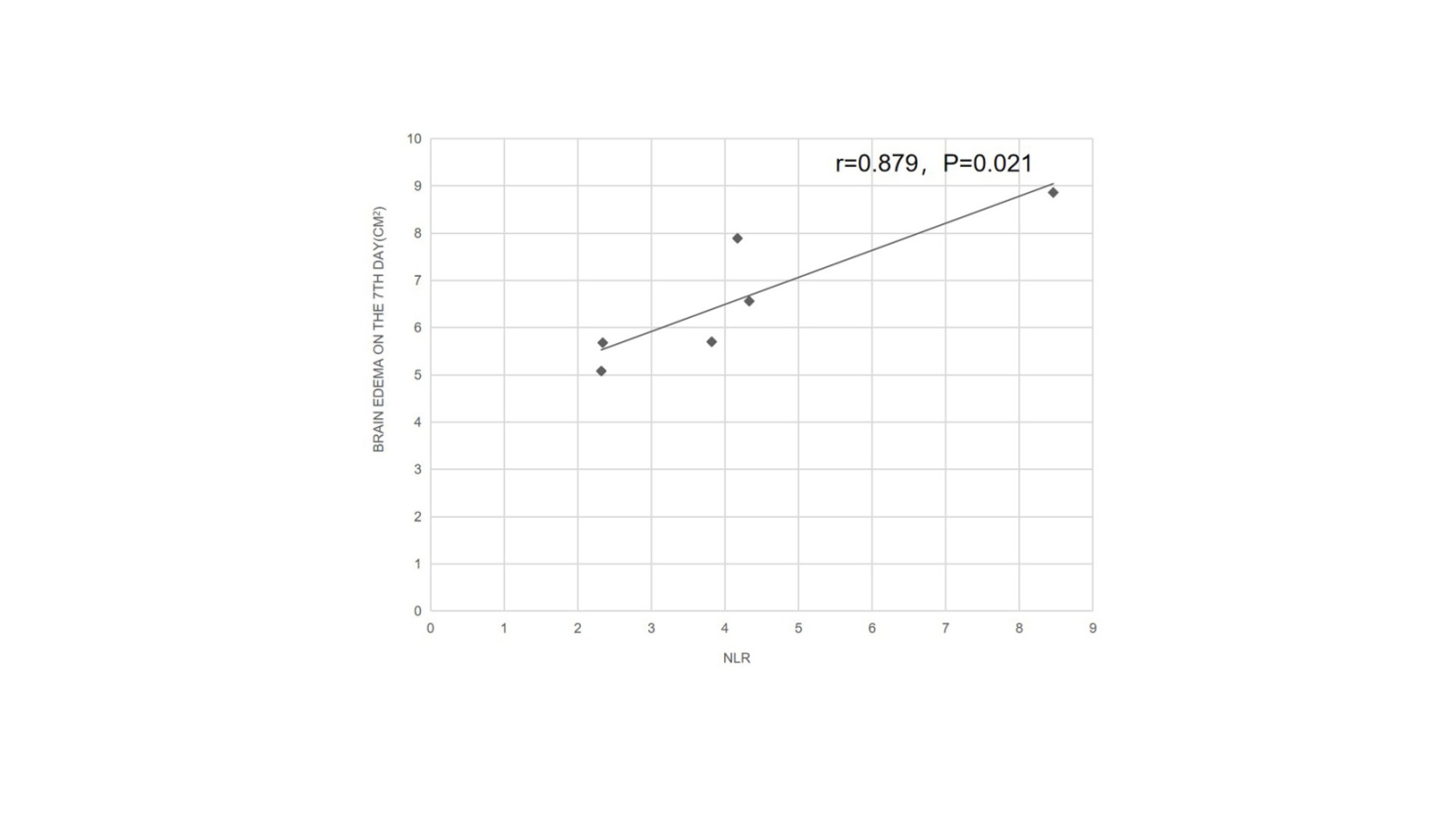


## Supplementary Figure 1. Baseline NLR levels may be related to brain edema area.
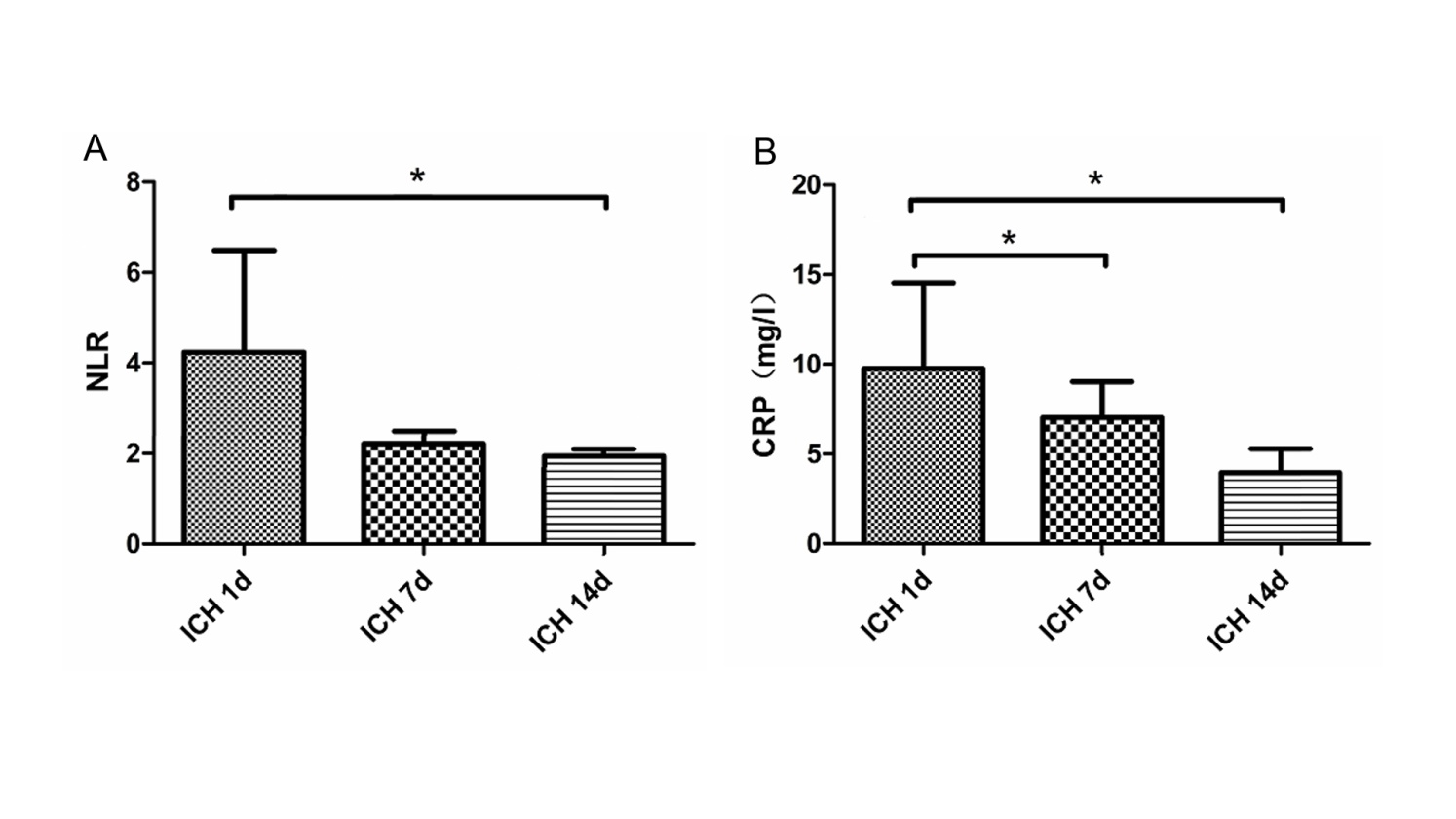


## Supplementary Figure 2. Changes of NLR (A) and CRP (B) on days 1, 7, and 14 after intracerebral hemorrhage. *P<0.05


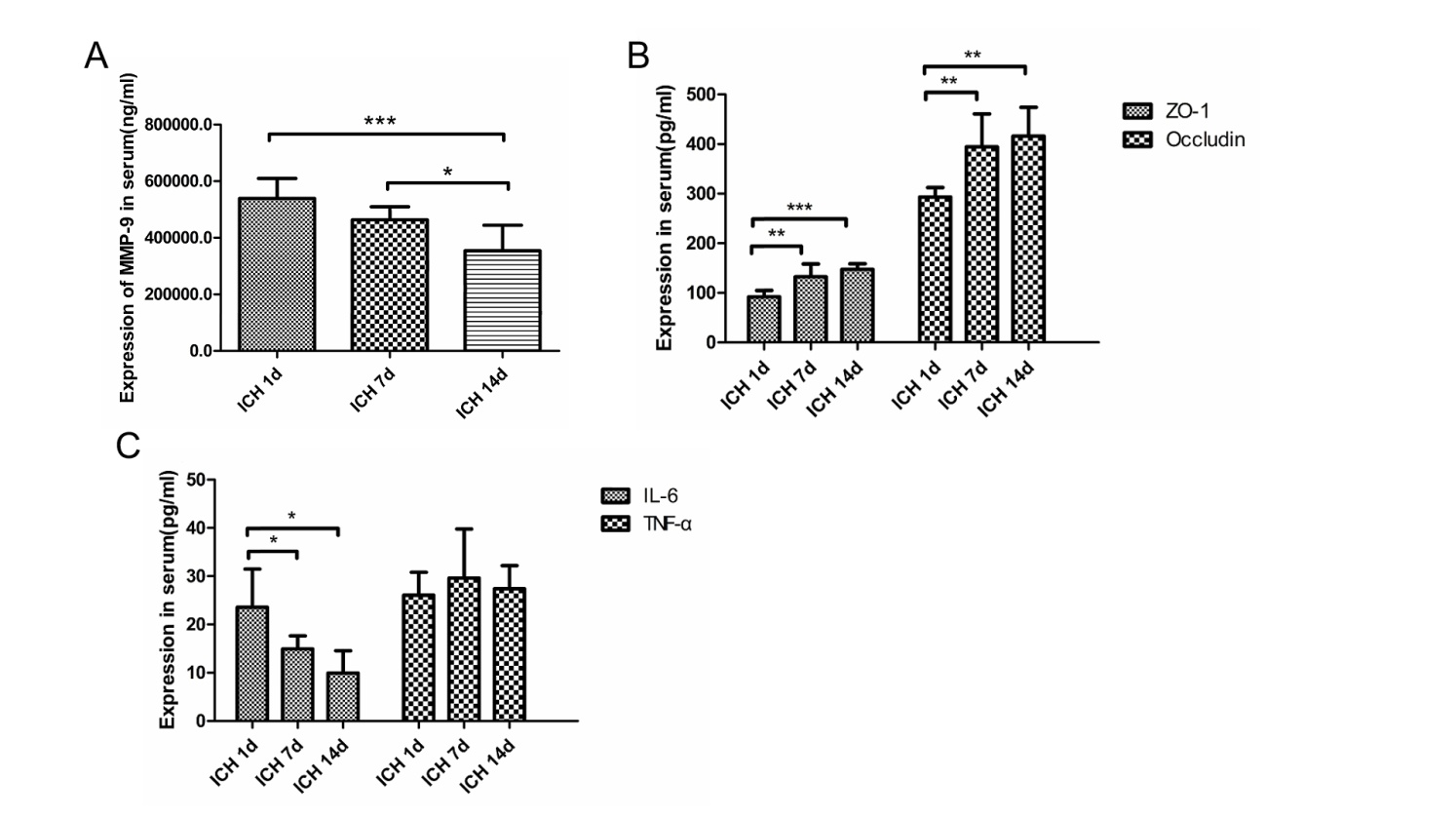


**Supplementary Figure 3.** The expression of MMP-9 (A)，ZO-1 (B)，Occludin (B)，IL-6 (C), and TNF-α (C) in the serum of patients with intracerebral hemorrhage at different time points. *P<0.05, **P<0.01, ***P<0.001
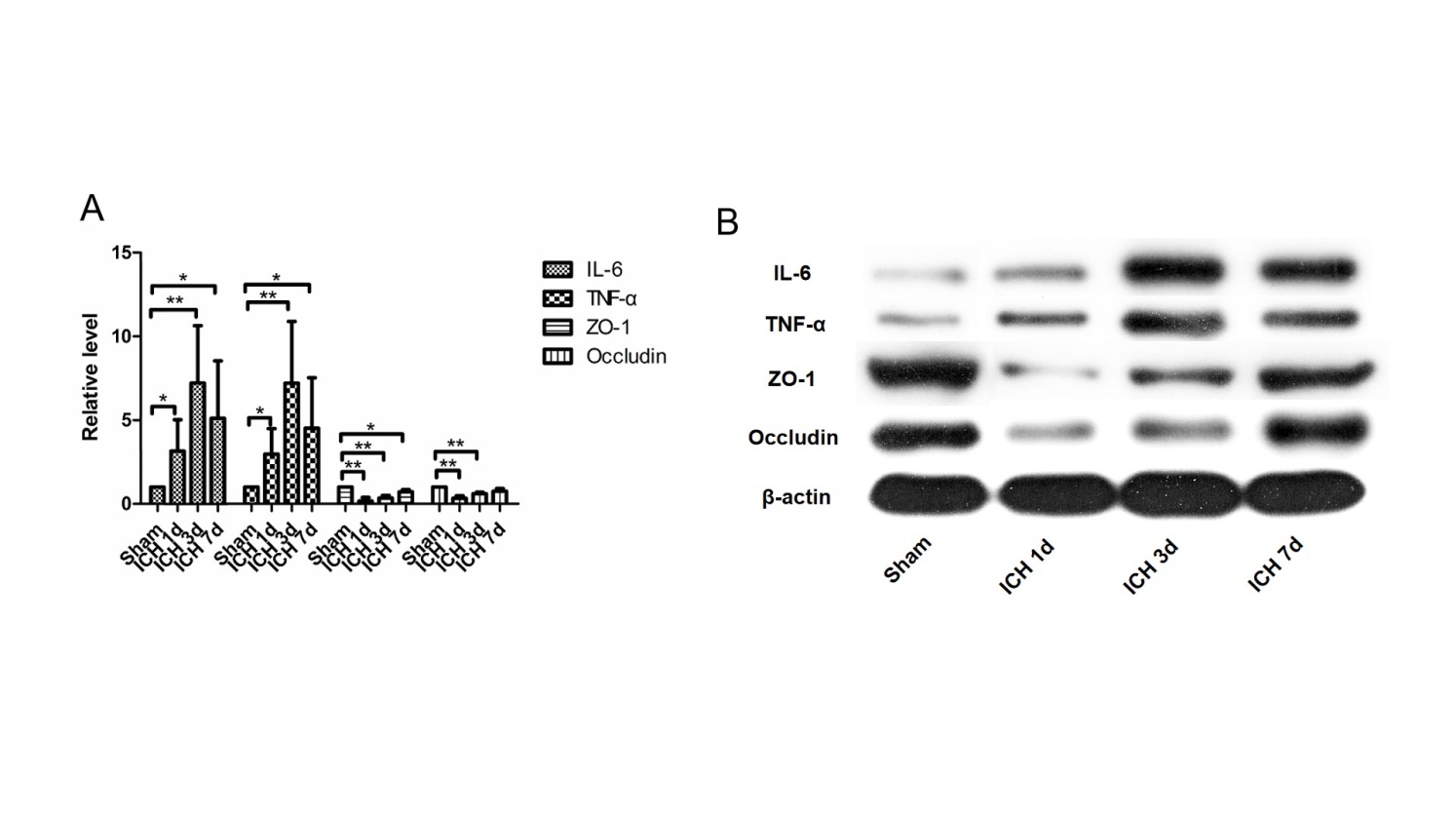
**Supplementary Figure 4.** IL-6 and TNF-αexpressions were increased after intracerebral hemorrhage，but ZO-1 and occluding expressions decreased after intracerebral hemorrhage. *P<0.05, **P<0.01


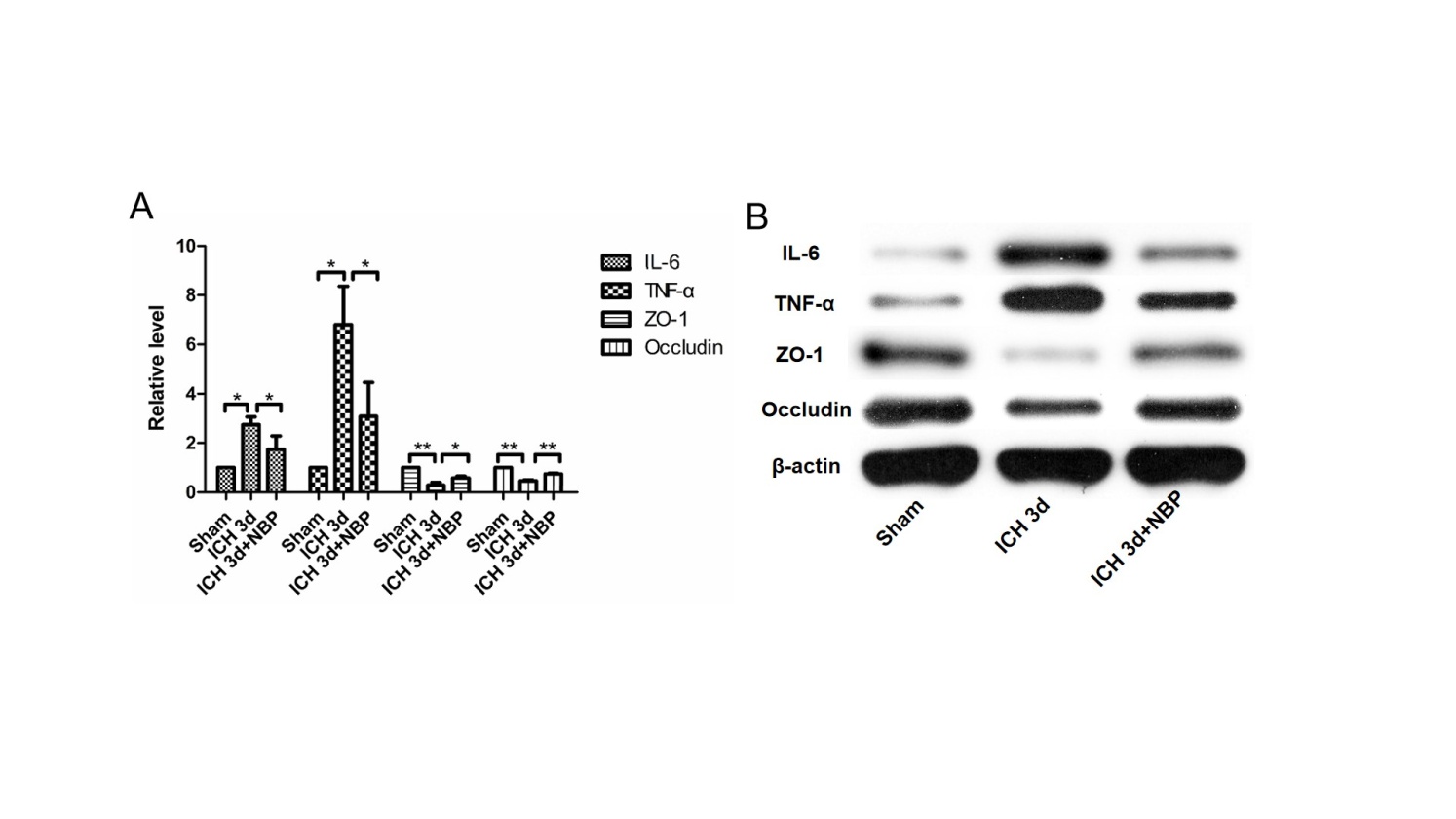
**Supplementary Figure 5.** IL-6 and TNF-a expressions decreased after the addition of NBP. While the tight junction proteins ZO-1 and occludin expressions increased after the addition of NBP. *P<0.05, **P<0.01
